# A facile and robust T7-promoter-based high-expression of heterologous proteins in *Bacillus subtilis*

**DOI:** 10.1186/s40643-022-00540-4

**Published:** 2022-05-18

**Authors:** Jing Ye, Yunjie Li, Yuqing Bai, Ting Zhang, Wei Jiang, Ting Shi, Zijian Wu, Yi-Heng P. Job Zhang

**Affiliations:** 1 Tianjin Key Laboratory of Food Science and Biotechnology, College of Biotechnology and Food Science, Tianjin University of Commerce, Tianjin, China; 2 Tianjin Institute of Industrial Biotechnology, Chinese Academy of Sciences, 32 West 7th Avenue, Tianjin Airport Economic Area, Tianjin, 300308 China

**Keywords:** *Bacillus subtilis*, T7 expression system, Recombinant protein expression, High cell-density fermentation

## Abstract

To mimic the *Escherichia coli* T7 protein expression system, we developed a facile T7 promoter-based protein expression system in an industrial microorganism *Bacillus subtilis*. This system has two parts: a new *B. subtilis* strain SCK22 and a plasmid pHT7. To construct strain SCK22, the T7 RNA polymerase gene was inserted into the chromosome, and several genes, such as two major protease genes, a spore generation-related gene, and a fermentation foam generation-related gene, were knocked out to facilitate good expression in high-density cell fermentation. The gene of a target protein can be subcloned into plasmid pHT7, where the gene of the target protein was under tight control of the T7 promoter with a ribosome binding site (RBS) sequence of *B. subtilis* (i.e., AAGGAGG). A few recombinant proteins (i.e., green fluorescent protein, α-glucan phosphorylase, inositol monophosphatase, phosphoglucomutase, and 4-α-glucanotransferase) were expressed with approximately 25–40% expression levels relative to the cellular total proteins estimated by SDS-PAGE by using *B. subtilis* SCK22/pHT7-derived plasmid. A fed-batch high-cell density fermentation was conducted in a 5-L fermenter, producing up to 4.78 g/L inositol monophosphatase. This expression system has a few advantageous features, such as, wide applicability for recombinant proteins, high protein expression level, easy genetic operation, high transformation efficiency, good genetic stability, and suitability for high-cell density fermentation.

**Graphical Abstract:**

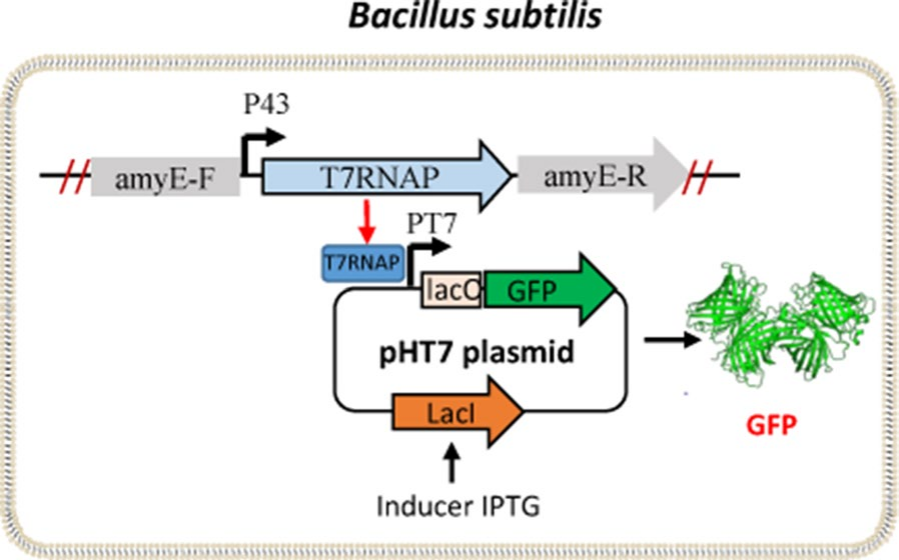

**Supplementary Information:**

The online version contains supplementary material available at 10.1186/s40643-022-00540-4.

## Introduction

The bacteriophage T7-protomer protein expression system is the most widely used technique of the production of recombinant proteins because of its simple genetic operation, high expression levels, and tightly regulated expression of targeted genes (Terpe 2006; Ting et al. 2020). It was first developed in the Gram-negative bacterium *Escherichia coli* (Rosenberg et al. 1987). The T7–*E. coli* expression system consists of two important parts: (1) an expression plasmid containing a gene of interest under the control of the T7 promoter and (2) a T7 expression host, such as *E. coli* DE3, which has a chromosomal copy of the T7 RNA polymerase gene with the control of a *lac*UV5 promoter. The isopropyl-β-D-thiogalactopyranoside (IPTG)-induced T7 expression can be regulated by co-expressing the *lac* repressor from the plasmid and by co-expressing the T7 lysozyme, a natural inhibitor of T7 RNA polymerase (Moffatt and Studier 1987). Later, this system has been adapted to other organisms, such as *Bacillus subtilis* (Conrad et al. 1996), *Bacillus megaterium* (Gamer et al. 2009), *Lactococcus lactis* (Wells et al. 1993), *Pseudomonas* (Davison et al. 1989), *Ralstonia eutropha* (Barnard et al. 2004), *Rhodobacter capsulatus* (Drepper et al. 2005), *Streptomyces lividans* (Lussier et al. 2010), *Shewanella oneidensis* (Yi and Ng 2021), and so on.

The Gram-positive bacterium *B. subtilis* is Generally Recognized As Safe (GRAS) microorganism due to its lack of pathogenicity and absence of endotoxins as well as its safe use as food and feed probiotics. It is one of the most important industrial hosts for the production of numerous proteins, especially homologous enzymes, such as α-amylase (Chen et al. 2015), protease (Dong et al. 2017; Wenzel et al. 2011), xylanase (Helianti et al. 2016; Rashid and Sohail 2021), lipase (Lu et al. 2010; Wu et al. 2020), β-glucanase (Niu et al. 2018), and so on (Schallmey et al. 2004; Terpe 2006). Also, a few heterogeneous enzymes have been over-expressed by *B. subtilis* by using different promoters (Harwood et al. 2002). For example, the natural strictly regulated xylose-inducible promoter P_xylA_ in *B. subtilis* has been demonstrated to achieve modest expression levels (Bhavsar et al. 2001; Kim et al. 1996). Similarly, several natural inducible promoters of *B. subtilis* have been investigated, such as P_glv_ (Yang et al. 2006; Yue et al. 2017), P_spaS_ (Bongers et al. 2005), P_gcv_ (Phan and Schumann 2007), and so on (Table 1). Furthermore, the heterologous P_spac_ promoter has been developed by fusing the 5ʹ-sequence of a promoter from the *B. subtilis* phage SPO1 and the 3ʹ-sequences of the *E. coli lac* promoter including its operator region and this promoter was inducible by a factor of 50 in terms of 1–10 mM IPTG (Yansura and Henner 1984). The P_grac_ promoter and its derived mutants based on the strong promoter of the *groESL* operon harboring the *lac* operator enabled the overexpression of beta-galactosidase to achieve up to 53% of the total cellular protein (Phan et al. 2012, 2006; Tran et al. 2020). However, most of them did not have as high expression efficiencies as those of the T7–*E. coli* system (i.e., ~ 15–50%) and/or suffered from low transformation efficiency or time-consuming genetic operations. Therefore, it is needed to develop a facile heterologous protein expression system in *B. subtilis*.

**Table 1:** The comparison of the protein expression systems of *B. subtilis* with different promoters

Promoter	Inducer	Characteristics	References
P_xylA_	Xylose	Strictly controlled by XylR repressor, and the protein expression was inhibited by glucose	(Bhavsar et al. 2001; Kim et al. 1996)
P_malA_	Maltose	Positively regulated by the transcriptional regulator MalR, and was repressed by glucose via CcpA and catabolite responsive elements	(Yang et al. 2006; Yue et al. 2017)
P_spaS_	Subtilin	The level of expression depended directly on the amount of inducer (subtilin) used, not subject to catabolite control	(Bongers et al. 2005)
P_gcv_	Glycine	The glycine tandem riboswitch was used to obtain regulatable expression of recombinant proteins	(Phan and Schumann 2007)
P_spac_	IPTG	Fused the 5ʹ-sequence of a promoter from the *B. subtilis* phage SPO1 and the 3ʹ-sequences of the *E. coli lac* promoter including its operator region	(Yansura and Henner 1984)
P_grac_	IPTG	Fused *groES* promoter and *lacO* operon and optimization of nucleotides at the conserved regions of the *groESL* promoter	(Phan et al. 2012, 2006; Tran et al. 2020)

Several efforts have been conducted to adapt the T7-promoter expression system into *B. subtilis.* The earliest attempt was conducted by Conrad et al. (Conrad et al. 1996). They designed an expression system composed of the T7 RNA polymerase under the control of xylose-inducible promoter P_xylA_ and the gene of interest under the control of the T7 promoter. In it, the T7 polymerase gene was inserted in the *amyE* site of the chromosome, and the genes of interest (i.e., α-amylase, β-1,4-glucosidase, and β-galactosidase) were inserted into the respective plasmids. However, the heterologous enzymes were expressed only when an antibiotic rifampicin was added to inhibit the host's inherent RNA polymerase. Recently, Sun and his coworkers further improved the T7 promoter system in an undomesticated *B. subtilis* strain ATCC6051a (Ji et al. 2021). The T7 RNA polymerase gene under the control of the P_xylA_ promoter was inserted in the *aprE* site chromosome for two purposes: to introduce the T7 RNA polymerase expression cassette and to break the native protease gene of the host. The expression frame of the target gene, which contains all the expression elements (i.e., T7 promoter, ribosome binding site sequence (RBS), the gene of interest, terminator) is highly similar to the pET21a vector, except that *E. coli* RBS (i.e., AAGGA) was replaced with the *B. subtilis* RBS sequence (i.e., AAGGAGG), and the whole expression frame was located in *E. coli–B. subtilis* shuttle vector pMK4 (Ji et al. 2021). To address the low transformation efficiency of *B. subtilis*, they inserted the *comK* gene responsible for the competence master regulator under the control of the xylose promoter in the *nprE* site of the chromosome (Zhang and Zhang 2011). They expressed approximately 1.0 g/L α-L-arabinofuranosidase in the LB media supplemented with 10 g/L xylose, which worked as both the inducer and carbon source. However, this system had two weaknesses, such as the genetic instability because the *comK* gene was ON in the presence of xylose because promoter P_xylA_ controlled its expression, and the inducer was consumed continuously by the host. Another strategy to address genetic instability was the insertion of both the T7 RNA polymerase gene and the gene of interest into the chromosome (Castillo-Hair et al. 2019; Chen et al. 2010). IPTG-inducible promoters (e.g., P_spac_ or P_hy-spank_) were used to regulate the expression of T7 RNA polymerase and integrated the genes of interest into the adjacent or distant site in the chromosome (Castillo-Hair et al. 2019; Chen et al. 2010). However, genetic modification of the chromosome was time-consuming and suffered from low biotransformation efficiency.

To develop a better T7-promoter expression system for *B. subtilis,* we need address several issues, such as easy biotransformation of the host, facile preparation of the expression plasmid, and a good expression host whose major proteases were knocked out. In this study, we developed a new *B. subtilis* host that featured (1) an inducible *comK* gene (Zhang and Zhang 2011); (2) the knock-out of some inherent protease genes, such as *aprE* and *nprE* (Kawamura and Doi 1984); (3) the insertion of the constitutive expression of the T7 RNA polymerase in the chromosome, and (4) the knock-out of the sporulation gene *spoIIAC* (Higgins and Dworkin 2012) and surfactin synthase gene *srfAC* (Peypoux et al. 1999) related to fermentation foam generation. The targeted gene was placed in an episomal plasmid pHT01 under the control of a hybrid promoter T7-lac and its ribosome binding site sequence was derived from *B. subtilis*. For any new targeted protein, the users can easily prepare the plasmid by one-step genetic operation (i.e., restriction enzyme-free and sequence-independent), prolonged overlap extension polymerase chain reaction (POE-PCR) (You et al. 2012) and easily transform the host with high transformation efficiencies. We tested heterologous expression of green fluorescent protein (GFP), α-glucan phosphorylase (αGP), inositol monophosphatase (IMP), phosphoglucomutase (PGM), and 4-α-glucanotransferase (4GT) proteins in *B. subtilis*, and demonstrated its applicability in high-density fermentation.

## Materials and methods

### Materials

All chemicals used were of analytical grade or higher quality and purchased from Sinopharm (Beijing, China), Aladdin (Shanghai, China), and Sigma-Aldrich (St. Louis, MO) unless specified. Taq DNA Polymerase was purchased from BioMed (Beijing, China), PrimeSTAR MAX DNA Polymerase and Premixed Protein Marker (Low) were purchased from Takara (Dalian, China). Plasmid extraction and PCR purification kit were purchased from Tiangen Biotech (Beijing, China).

### Strains, plasmids, and cultivation conditions

Strains and plasmids used are listed in Table 2. Plasmids were constructed using the Simple Cloning technology (You et al. 2012). The primers (Table 3 and Additional file 1: Table S1) used for PCR were synthesized by GENEWIZ (Beijing, China). *B. subtilis* SCK6 which was derived from *B. subtilis* 1A751 contains a genetic cassette expressing the *comK* gene in its genome (Zhang and Zhang 2011). An *E. coli–B. subtilis* shuttle vector pHT01 (Nguyen et al. 2007) was used to clone and express the desired recombinant protein. The strains were cultured in Luria–Bertani (LB) medium containing 0.5% yeast extract, 1% tryptone, and 1% NaCl at 37 °C. When necessary, the medium was supplemented with 5 mg/L chloramphenicol or 0.3 mg/L erythromycin.

**Table 2:** Bacterial strains and plasmids

Strain or plasmid	Characteristics	Source
Bacillus subtilis		
SCK6	Erm^R^, his *nprR2 nprE18* △*aprA3* △*eglS102* △*bglT bglSRV*, *lacA*::P_*xylA*_*-comK*	Lab stock
SCK8	Erm^R^, SCK6 derivate, △*upp*	This work
SCK9	Erm^R^, SCK8 derivate, △*upp*△*spoIIAC*	This work
SCK10	Erm^R^, SCK9 derivate, △*upp*△*spoIIAC*△*srfAC*	This work
SCK22	Erm^R^, SCK10 derivate, △*upp*△*spoIIAC*△*srfAC*, *amyE*::P_43_-T7RNAP	This work
Plasmids		
pSS	Amp^R^, Cm^R^, modular vector carrying *upp*-cassette	Lab stock
pHT01	Amp^R^, Cm^R^, *E. coli-B. subtilis* shuttle vector	Lab stock
pDG1730	Spc^R^, integration vector contains a spectinomycin resistance gene sandwiched between *amyE*-front and *amyE*-back	Lab stock
pDG1730-T7RNAP	Spc^R^, pDG1730 derivate, used to integrate genes on the genome, with T7RNAP expression cassette (*spc*-*upp*-P_43_-T7RNAP) and *amyE* gene upstream and downstream homology arms	This work
pHT7	Amp^R^, Cm^R^, pHT01 derivate, with T7-lac promoter	This work
pHT7-GFP	Amp^R^, Cm^R^, pHT7 derivate, with GFP cloned	This work
pHT7-αGP	Amp^R^, Cm^R^, pHT7 derivate, with αGP cloned	This work
pHT7-IMP	Amp^R^, Cm^R^, pHT7 derivate, with IMP cloned	This work
pHT7-PGM	Amp^R^, Cm^R^, pHT7 derivate, with PGM cloned	This work
pHT7-4GT	Amp^R^, Cm^R^, pHT7 derivate, with 4GT cloned	This work

**Table 3:** Primers used in this work

Primers	Oligo sequences (5ʹ → 3ʹ)
Primers used in construction of pHT7-GFP	
P1	gtttaactttaagaaaggaggatataccatgagtaaaggagaagaacttttc
P2	gctttgttagcagccggatctcattatttgtatagttcatccatgccatg
P3	catggcatggatgaactatacaaataatgagatccggctgctaacaaagc
P4	gaaaagttcttctcctttactcaggtatatcctcctttcttaaagttaaac
Primers used in construction of pHT7-αGP	
P5	ctttaagaaaggaggatataccatggtgaacgtttccaatgccgttgaggatg
P6	cgggctttgttagcagccggatcttagtcaagtcccttccacttgaccagac
P7	catcctcaacggcattggaaacgttcaccatggtatatcctcctttcttaaag
P8	gtctggtcaagtggaagggacttgactaagatccggctgctaacaaagcccg
Primers used in construction of pHT7-IMP	
P9	taactttaagaaaggaggatataccatgctggatcgcctggatttctctattaaactgctgcg
P10	cgggctttgttagcagccggatctcatttaccgccgatttcttcaacaac
P11	taactttaagaaaggaggatataccatgctggatcgcctggatttctctattaaactgctgcg
P12	gttgttgaagaaatcggcggtaaatgagatccggctgctaacaaagcccg
Primers used in construction of pHT7-PGM	
P13	ctttaagaaaggaggatataccatggggaagctgtttggaacatttggag
P14	cgggctttgttagcagccggatcttatgaaagcgctttctcaagtagctc
P15	gagctacttgagaaagcgctttcataagatccggctgctaacaaagcccg
P16	ctccaaatgttccaaacagcttccccatggtatatcctcctttcttaaag
Primers used in construction of pHT7-4GT	
P17	ctttaagaaaggaggatataccatggaacgtatcaacttcatcttcggtatcc
P18	ggctttgttagcagccggatctcacagttcgcgaaaacgaacggtgaattcc
P19	ggaattcaccgttcgttttcgcgaactgtgagatccggctgctaacaaagcc
P20	ggataccgaagatgaagttgatacgttccatggtatatcctcctttcttaaag

### Construction of the *B. subtilis* SCK22 strain

**Fig. 1:**
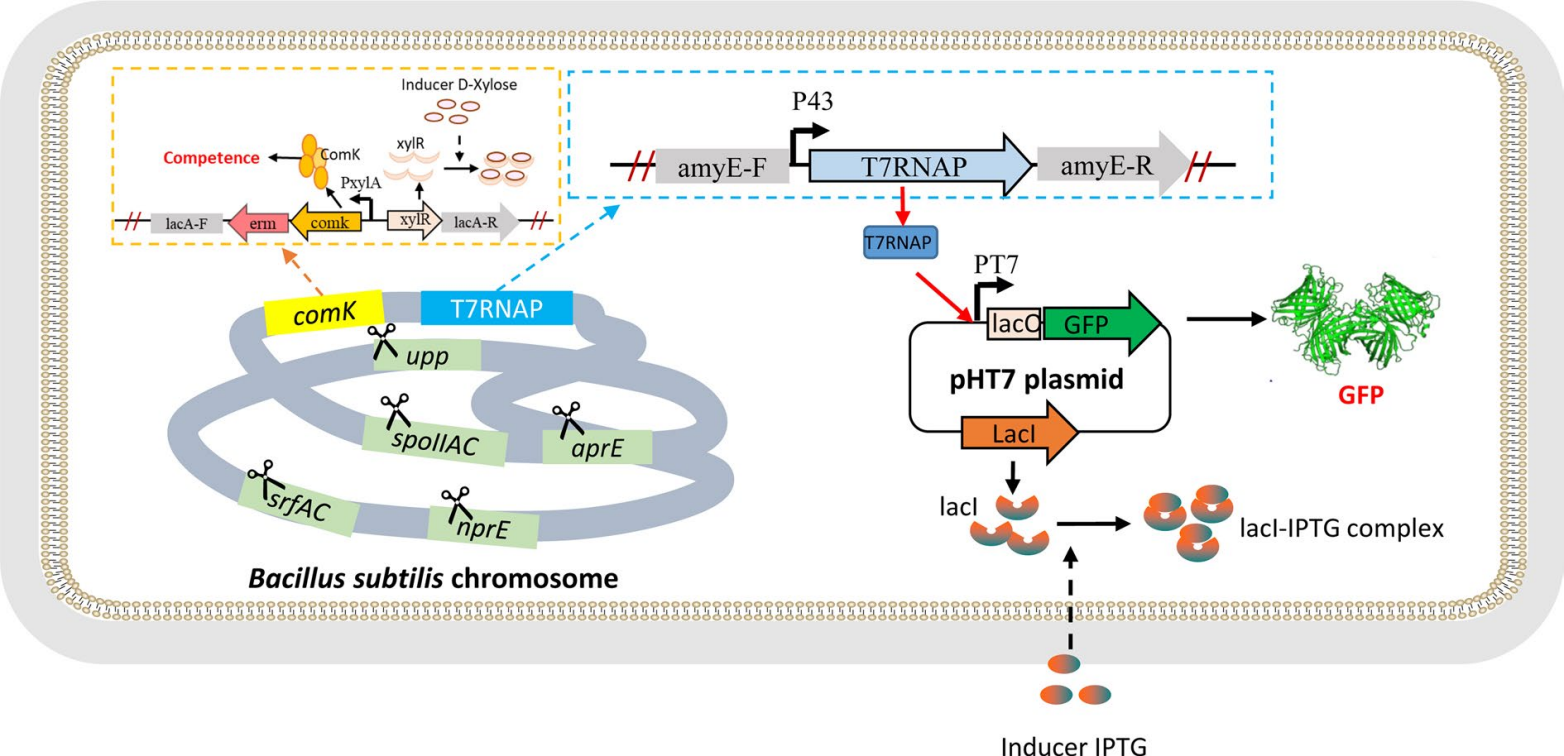
Schematic representation of the industrial host SCK22 harboring plasmid pHT7. After xylose induction, under the control of xylose-inducible promoter, *comK* gene was expressed and then super receptive cells were formed. Endogenous protein genes and fermentation-related genes were knocked out on the genome to make it suitable for high-density fermentation. The P_43_ promoter enabled the constitutive expression of T7 RNA polymerase, and the expression plasmid encoded the target gene, where the T7 promoter was under the control of the *lac* operon

Figure 1 shows the design of the T7 expression system in *B. subtilis*. To construct a seamless knock-out system, the *upp* gene was knocked out as a negative selection marker gene. The *upp* gene encodes uracil phosphoribosyltransferase (UPRTase), which can catalyze pyrimidine analog 5-fluorouracil (5-FU) to 5-fluoro-dUMP, which is a strong inhibitor of thymidylate synthetase and leads to cell death. Deletion of *upp* endows the mutant strain with resistance to 5-FU (Dong and Zhang 2014).

The knock-out of a gene in the chromosome was conducted by double-crossover homologous recombination (Shi et al. 2013). In brief, the upstream homology arm of the target gene including the direct repeat DR region, the *upp* gene, the antibiotic gene, and the downstream homology arm of the target gene including the DR region were sequentially connected to form large-size DNA multimers by prolonged overlap extension PCR and then were transferred into *B. subtilis*. Primer sequences used for PCR are in the supplementary materials (Additional file 1: Table S1).The first-round double- crossover homologous recombination occurred with the *upp* gene and antibiotic gene integrated into the chromosome through resistance plate screening. The correct transformants verified by PCR were cultured in the resistance-free medium, and the second-round homologous recombination occurred between the two DR regions. Thus, the target transformants, where the *upp* gene, resistance gene and the target gene were all deleted, could be obtained by using 5-FU plate screening.

For strains SCK8, SCK9 and SCK10, the *upp*, *spoIIAC* and *srfAC* genes were knocked out by using this seamless knock-out system, respectively. The pSS plasmid backbone, upstream homology arm of the target gene, *upp* gene fragment, chloramphenicol resistance gene fragment, and downstream homology arm gene of the target gene were sequentially connected to obtain an integration plasmid by prolonged overlap extension PCR (Morimoto et al. 2008; Shi et al. 2013). Subsequently, the plasmid was transferred into *B. subtilis* and the target transformants were obtained by two rounds of homologous recombination (Shi et al. 2013).

SCK22 was constructed based on the mutant strain SCK10. The integration vector pDG1730 was used as it contained a spectinomycin resistance gene sandwiched between *amyE*-front and *amyE*-back (Guerout-Fleury et al. 1996). The pDG1730 plasmid backbone, *upp* gene fragment, the upstream homology arm DR region and a DNA cassette encoding the T7 RNA polymerase with the P_43_ promoter were sequentially connected to obtain a new plasmid pDG1730-T7RNAP by using Simple Cloning (You et al. 2012). Then, the constructed integrated plasmid pDG1730-T7RNAP was transformed into *B. subtilis* SCK10 to obtain SCK22.

### Construction of the pHT7-based expression vectors

**Fig. 2:**
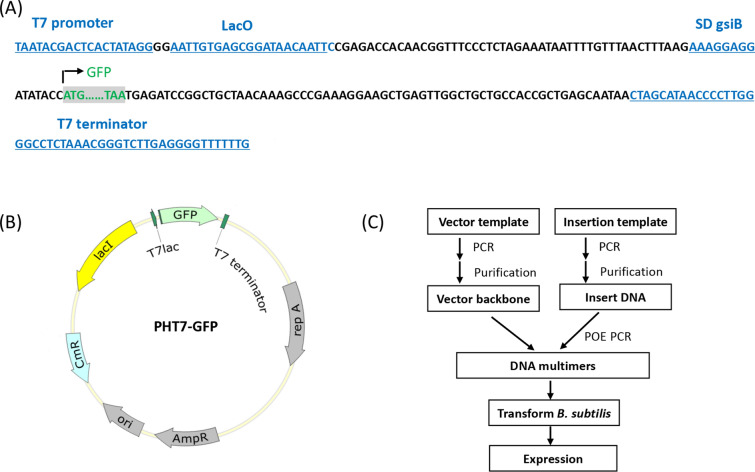
Key DNA sequence before and after the gene of interest (**A**), plasmid map of pHT7-GFP (**B**) and scheme of plasmid multimerization (**C**). Plasmid pHT7-GFP contains the T7 promoter-terminator cassette including a *lacO* lactose operator and a SD sequence, the replication origin *ori* in *E. coli*, the replication origin *repA* in *B. subtilis*, the *lac* repressor gene, chloramphenicol-resistance gene and ampicillin-resistance gene

The DNA fragment containing a T7-lac promoter, T7 terminator, and the *B. subtilis* RBS sequence (i.e., AAGGAGG) (Fig. 2A) was chemically synthesized. This sequence was subcloned into plasmid pHT01, yielding plasmid pHT7. The *gfp* gene derived from *Aequorea victoria* was selected as the gene of interest and inserted after the RBS sequence of the pHT7 plasmid (Fig. 2A and B). The insertion DNA fragment encoding green fluorescent protein (GFP) was amplified by PCR with a pair of primers P1 and P2 (Table 3). The linear vector backbone was amplified from plasmid pHT7 with a pair of primers P3 and P4. The two PCR products were assembled by POE‐PCR. The POE‐PCR product was directly transformed into *B. subtilis* SCK22, yielding plasmid pHT7-GFP. The plasmid pHT7-GFP was used as the protein expression vector to verify and optimize the newly constructed T7 expression system. The other four enzyme expression plasmids (i.e., α-glucan phosphorylase (αGP) from the thermophilic bacterium *Thermococcus kodakarensis,* inositol monophosphatase (IMP) from *Thermotoga maritima*, phosphoglucomutase (PGM) from *T. kodakarensis*, and 4-α-glucanotransferase (4GT) from *Thermococcus litoralis*) were constructed in the same way (Fig. 2C). The gene sequences of these four enzymes were described elsewhere (You et al. 2017; Zhou et al. 2016).

### Transformation of the *B. subtilis* SCK22 strains

The transformation of *B. subtilis* SCK22 was performed as described elsewhere (Zhang and Zhang 2011) with minor modifications. The *B. subtilis* SCKC22 strain was spread on solid LB medium containing 0.3 mg/L erythromycin and then cultured overnight in 37 °C. Single colonies were picked from the plate and then inoculated in 3 mL of the LB liquid medium containing 0.3 mg/L erythromycin at 37 °C. The cell cultures were incubated in a 250 rpm shaker for 4 h. The cultures were then transferred to 50 mL of the LB medium and grew at 37 °C until the absorbency at 600 nm was approximately 0.6–0.8. Xylose (final concentration of 10 g/L) was added and cultured for 2 h. The resulting cell cultures were ready to be transformed as super-competent cells or divided into aliquots and stocked at − 80 °C with 10% (v/v) glycerol for future use (thawed for direct transformation). Then 1 μL of POE-PCR product was mixed with 100 μL of the super-competent cells, and then was incubated in a rotary shaking incubator at 200 rpm for 1.5 h at 37 °C. Spread the transformed competent cells on solid LB plate with the appropriate antibiotic and incubate the plate at 37 °C for 8–12 h to select transformants.

### Heterologous protein expression

The SCK22 strains containing plasmids pHT7-GFP, pHT7-αGP, pHT7-IMP, pHT7-PGM or pHT7-4GT were cultured in small culture tubes overnight and then inoculated into 1-L shake flasks containing 200 mL of the LB liquid medium at 37 °C. The inoculum size was adjusted to allow the cell culture to have an absorbency of about 0.05 at 600 nm. When A_600_ was reached 0.8–1.0, IPTG was added, followed by 4 h of cell cultivation. After fermentation, the broth was centrifuged. The cell pellets were washed with the saline water once. The cell pellets were re-suspended in 50 mM HEPES buffer (pH 7.0) containing 100 mM NaCl. After ultra-sonication and centrifugation, the supernatants containing all soluble proteins including the target protein were analyzed by SDS-PAGE according to the standard procedure. The gels were stained by Coomassie brilliant blue R250 staining.

### Fluorescence measurement and quantification of GFP

Cell cultures of SCK22/pHT7-GFP were centrifuged at 12,000×*g* for 5 min to obtain bacterial cells and supernatants. After the bacterial cells were washed with the saline water once, they were re-suspended in the 50 mM HEPES buffer (pH 7.0) containing 100 mM NaCl prior to disruption by ultra-sonication on an ice bath. The fluorescence intensities of the supernatants of cell lysates and fermentation broth represented the intracellular and extracellular GFP concentrations, respectively. The expression level of GFP protein was calculated according to the measured fluorescence intensity and fluorescence curve (Additional file 1: Figure S1). Cell growth was monitored by measuring its absorbance at 600 nm.

### Fed-batch fermentation

The fermentation was carried out in a 5-L fermenter (T&J Bio-engineering Co., Shanghai, China). The fermentation medium consisted of the following components (per liter): 10 g of yeast extract, 0.2 g histidine, 20 g glycerol, 5.12 g Na_2_HPO_4_·12H_2_O, 3.0 g KH_2_PO_4_, 0.5 g NaCl, 0.5 g MgSO_4_·7H_2_O, 0.011 g CaCl_2_, 1.0 g NH_4_Cl, 0.2 mL of 1% (w/v) vitamin B1, and 0.1 mL of the trace elements solution. The stock solution of trace elements contained the following (per liter) in 3 M HCl: 80 g FeSO_4_·7H_2_O, 10 g AlCl_3_·6H_2_O, 2.0 g ZnSO_4_·7HO, 1.0 g CuCl_2_·2H_2_O, 2.0 g NaMoO_4_·2H_2_O, 10 g MnSO_4_·H_2_O, 4.0 g CoCl_2_, and 0.5 g H_3_BO_4_. Appropriate antibiotics and defoamer were added if necessary.

The cryopreserved strains were inoculated into 50 mL of the LB medium containing 1% glucose and then cultured at 37 °C for 12 h with vigorous shaking. Then the entire cell cultures were transferred into the fermenter. Dissolved oxygen (DO) was monitored using a DO sensor and was maintained above 20% saturation by controlling both the aeration rate (2–18 L/min) and the agitation rate (200–1000 rpm). Foaming was controlled by the addition of the Sigma anti-foaming agent. After about 8 h cultivation, the DO shown a suddenly increased, indicating the complete consumption of carbon source. The feeding solution (i.e., 50% (g/g) glycerol, 5% (g/g) yeast extract, and 0.5% (g/g) histidine) was added slowly. The addition rate of the feeding solution was adjusted to be approximately 6–10 g/L/h according the growth rates of bacteria. The fermentation was performed at pH 6.8 and 37 °C, whereas the pH was adjusted with 25% (v/v) ammonia. The samples were collected at the indicated time intervals.

### Protein analysis by SDS-PAGE

Cell culture samples were harvested and centrifuged at 12,000×*g* for 5 min. The pellets were re-suspended in 50 mM HEPES buffer (pH 7.0) containing 100 mM NaCl. After ultra-sonication in an ice bath, cell debris were removed by centrifugation at 12,000×*g* for 5 min. After adding the SDS-PAGE loading buffer, the cell lysates and the supernatants of the cell lysates were incubated in a boiling water bath for 10 min and equal amounts of proteins were loaded into 12% SDS-PAGE gels. The Premixed Protein Marker (Low covering the 14.3 to 97.2 kDa range) (Takara Bio Inc., China) was used as a molecular mass marker. Following electrophoresis, proteins were visualized by Coomassie Brilliant Blue R250. The SDS-PAGE results were imaged and analyzed by Bio-Rad Gel Doc™ XR + Imaging System.

### Other assays

The concentrations of the proteins were determined by the Bradford with bovine serum albumin as the reference. All data were averaged from three independent samples.

## Results

### Construction of the T7 expression system in *B. subtilis*

Figure 1 shows the design of the *Bacillus* T7 expression system. Similar to the *E. coli* T7 system, it had two parts: a plasmid encoding the gene of interest which was under control of the T7 promoter, and a *Bacillus* host whose chromosome had a T7 RNA polymerase gene under the control of a constitutive P_43_ promoter. First, because *B. subtilis* has much lower transformation efficiency than *E. coli* and it was time-consuming to prepare competent cells, the DNA cassette containing the *comK* gene under the control of the inducible P_xylA_ promoter was inserted into its chromosome, wherein the ComK of *B. subtilis* was the master regulator for competence development (Mironczuk et al. 2008; Zhang and Zhang 2011). The induced super-competent cells of *B. subtilis* exhibited transformation efficiencies of ~ 10^7^ transformants per μg of multimeric plasmid DNA (Zhang and Zhang 2011). Second, similar to the *omp*T- and *ion*-deficient *E. coli* BL21, two *Bacillus* protease genes (i.e., *aprE* and *nprE*) were knocked out from the chromosome so that the host was suitable for the expression of recombinant protein. Third, to avoid sporulation during its fermentation, the *spoIIAC* gene (RNA polymerase sigma-F factor) was knocked out (Zhang et al. 2016)*.* Fourth, the surfactin synthase gene *srfAC* (surfactin synthase subunit 3) was also knocked out because its expression could form broth foam, impairing high-density fermentation (Zhang et al. 2016). Last, the T7 RNA polymerase gene with its P_43_ promoter was inserted into the *amyE* gene of the chromosome, yielding the T7 expression host *B. subtilis* SCK22.

Expression plasmid pHT7 (Fig. 2) was constructed to contain a T7-*lac* hybrid promoter, *B. subtilis* RBS sequence (i.e., AAGGAGG), the gene of interest (e.g., green fluorescent protein, *gfp*) and the T7 terminator, based on pHT01 plasmid. The partial DNA sequence of plasmid pHT7 before and after the gene of interest is presented in Fig. 2A. In the absence of the inducer IPTG, a repressor protein (LacI) that repressed T7-*lac* promoter transcription prevented the target gene from being synthesized. When IPTG was added, it would bind to LacI and release the tetrameric repressor from the *lac* operator, thereby allowing the transcription of the T7-*lac* operon followed by the synthesis of a large amount of the targeted protein.

### Flask fermentation and optimization

**Fig. 3:**
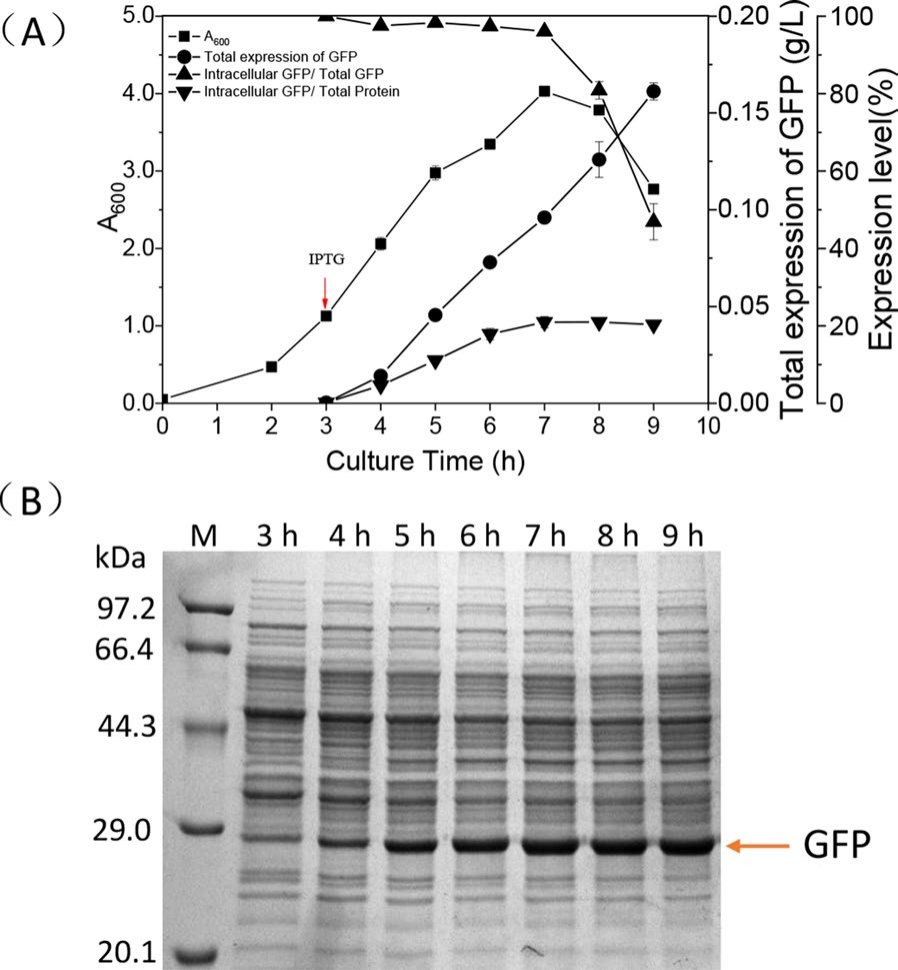
Profile of fermentation of *B. subtilis* SCK22/pHT7-GFP (**A**) and SDS-PAGE analysis of GFP over time (**B**)

The strain SCK22 harboring plasmid pHT7-GFP was cultured in 1-L flasks, wherein the amount of green fluorescent proteins could be quantitated by measuring the fluorescence intensity of GFP. The profile of the fermentation of strain SCK22/pHT7-GFP is shown in Fig. 3A. The absorbency of cells at A_600_ rose to 4.1 at the 7th hour and then declined slowly. When the A_600_ was about 1.0, 0.5 mM IPTG was added. GFP was synthesized after IPTG addition and its concentration kept increasing to 0.16 g/L. After 4-h induction, nearly all GFP was intracellular and the GFP content relative to its total cellular protein was approximately 21%. Because some fraction of cells began to lyse after reaching the highest cell density, approximately a third GFP was released to the broth at the end of fermentation. SDS-PAGE analysis also shows the increased GFP expression over time (Fig. 3B). The intracellular GFP content gradually increased to 19.6% after IPTG addition until it reached the maximum after 4 h.

**Fig. 4:**
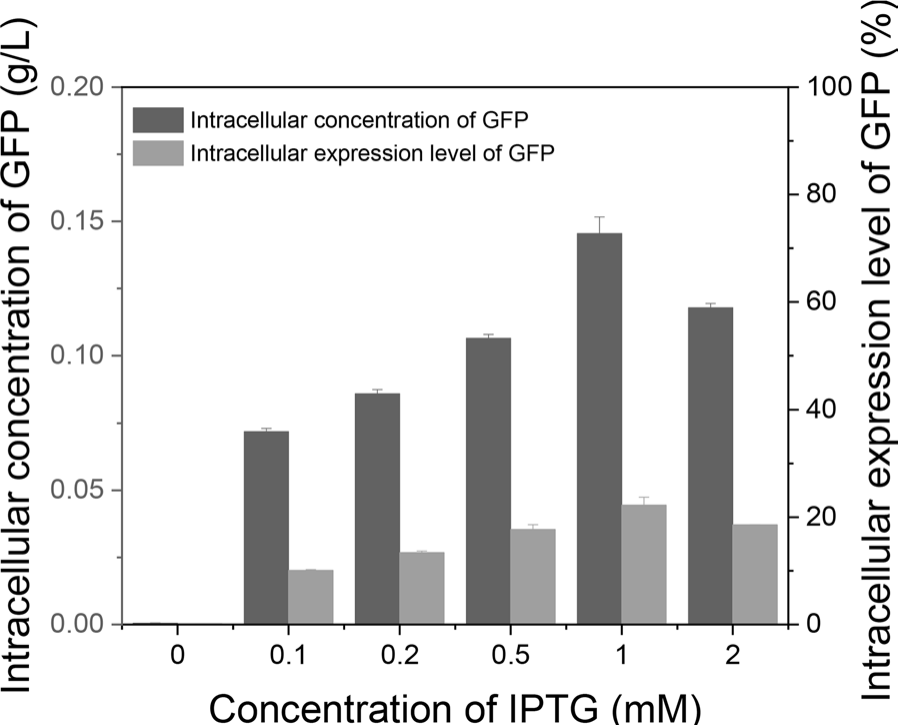
Effects of the inducer IPTG concentration on GFP expression level and total GFP concentration

We also tested the optimal IPTG concentration from 0 to 2.0 mM (Fig. 4). The maximum intracellular GFP concentration (0.146 g/L) was obtained when IPTG was 1.0 mM. The GFP content was 22.4% relative to the total cellular protein.

### Synthesis of four other heterologous proteins

**Fig. 5:**
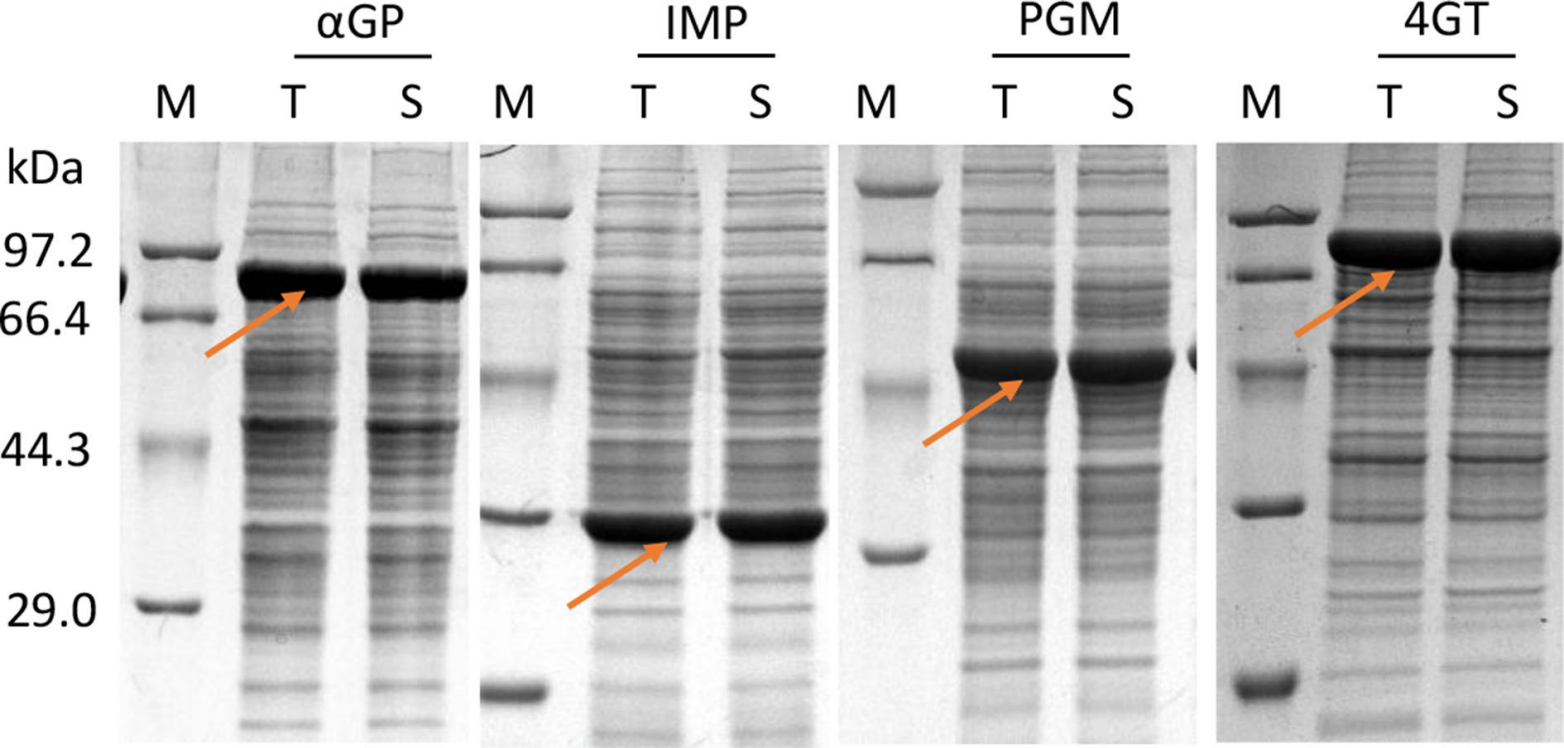
SDS-PAGE analysis of targeted protein expression in *B*. *subtilis* SCK22. Lanes: *M* the standard protein markers, *T* the total cell lysate, *S* the supernatant of the total cell lysate, *αGP* α-glucan phosphorylase, *IMP* inositol monophosphatase, *PGM* phosphoglucomutase, *4GT* 4-α-glucanotransferase

We tested the applicability of this expression to four other proteins. They were αGP from *T. kodakarensis*, IMP from *T. maritima*, PGM from *T. kodakarensis*, and 4GT from *T. litoralis*. These thermophilic enzymes were used to synthesize inositol from starch in vitro (You et al. 2017; Zhou et al. 2016). Their expression was initiated by adding 1.0 mM IPTG when A_600_ reached approximately 0.8–1.0. SDS-PAGE (Fig. 5) shows that the expression levels of αGP, IMP, PGM, and 4GT were 31.7%, 26.3%, 24.3%, and 40.3%, respectively. There were no inclusion bodies found for all cases (Fig. 5), which was confirmed by the measuring the difference of protein concentrations in the cell lysate and its supernatants. These results suggested that this *Bacillus* T7 expression system can be used to express numerous heterologous proteins efficiently.

### Fed-batch high-cell-density fermentation

**Fig. 6:**
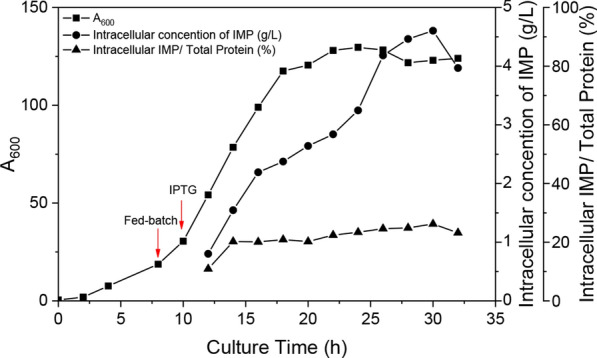
Profile of high-cell-density fermentation of *B. subtilis* SCK22/pHT7-IMP in a 5-L fermenter

To investigate whether the newly constructed T7 expression system is suitable for high-density fermentation, *B. subtilis* SCK22/pHT7-IMP was tested in fed-batch fermentation. As shown in Fig. 6, after 8 h fermentation, the feeding solution was added. With the feed addition, the cell concentration continued to increase (A_600_ up to 129.6). When 1.0 mM IPTG was added at 10 h, the IMP was synthesized. After 30 h fermentation, the intracellular IMP expression level reached a peak, accounting for 27.2% of the total intracellular protein, and the IMP titer was 4.78 g/L. Afterwards, the intracellular expression level of IMP declined due to cell lysis. SDS-PAGE analysis was also conducted to obtain of the relative percentage of intracellular IMP to the total cellular protein (Additional file 1: Figure S2). These results showed that this *Bacillus* T7 expression system was also suitable for high-density fermentation.

## Discussion

In this study, we developed a simple *Bacillus* T7 protein expression system. This system contained a *B. subtilis* SCK22 host recombinant strain and a plasmid pHT7. The host was deficient in two major protease genes (i.e., *aprE* and *nprE*), a sporulation gene and (*spoIIAC*), and a surfactin synthase genes *srfAC*. Two genes were inserted its chromosome: the xylose-inducible *comK* gene for high transformation and the constitutive T7 RNA polymerase gene. With an available *B. subtilis* SCK22, it was easy and fast to construct the expression plasmid by using POE-PCR and transform into the host with high transformation efficiency. Five heterologous proteins were expressed efficiently in this system. As compared to other *Bacillus* T7-derived expression systems (Castillo-Hair et al. 2019; Chen et al. 2010; Conrad et al. 1996; Ji et al. 2021), this system featured its wide applicability, easy genetic operation, high expression capacity in both flask and fed-batch fermentation, and tightly controlled synthesis of the target protein.

It was notable that this *Bacillus* T7 expression synthesis could be superior to the *E. coli* counterpart for some proteins. It found out that at least a half of recombinant 4GT synthesized was inclusion bodies when it was expressed in *E. coli* (Additional file 1: Figure S3) although its expression conditions were intensively optimized, for example, decreased protein synthesis temperature, various IPTG concentration, codon optimization, co-expression of multiple chaperones (Duan et al. 2019). In contrast, there was not a significant amount of inclusion body observed when it was expressed in *Bacillus*. The reasons behind the better protein synthesis and folding in the *Bacillus* T7 expression could be under further investigation.

## Conclusion

In conclusion, to develop a better T7-promoter expression system for *B. subtilis*, the strain SCK22 with high transformation efficiency and suitable for high-density fermentation was constructed by double-crossover homologous recombination, and the plasmids were constructed easily by simple cloning. The intracellular expression level of heterologous proteins reached the highest level of 25% ~ 40% at 4 h after 1.0 mM IPTG induction. The yield of IMP reached 4.78 g/L in high-density fermentation. In summary, the *Bacillus* T7 expression system has the advantages of simple genetic operation, stable expression of heterologous proteins, wide applicability, and suitable for high-density fermentation.

### Supplementary Information

**Additional file1:** **Figure S1. **The standard curve of GFP protein concentration and its fluorescence intensity. **Figure S2.** SDS-PAGE analysis of IMP of *B. subtilis* SCK22/pHT7-IMP in a 5-L fermenter over time. Lanes: M, protein markers; T, the cell lysate; S, the supernatant of the cell lysate. **Figure S3.** SDS-PAGE analysis of expression of 4-α-glucanotransferase (4GT) from *Thermococcus litoralis* in *E.coli* BL21(DE3). Lanes: M, protein markers; T, the cell lysate; S, the supernatant of the cell lysate. **Table S1. **Primers for gene knockout.

## Data Availability

The datasets supporting this article are included in the manuscript.

## Abbreviations

RBS: Ribosome binding site
SDS: Sodium dodecyl sulfate
PAGE: Polyacrylamide gel electrophoresis
IPTG: Isopropyl-β-D-thiogalactopyranoside
GRAS: Generally recognized as safe
POE-PCR: Prolonged overlap extension polymerase chain reaction
GFP: Green fluorescent protein
αGP: α-Glucan phosphorylase
IMP: Inositol monophosphatase
PGM: Phosphoglucomutase
4GT: 4-α-Glucanotransferase
LB: Luria–Bertani
UPRTase: Uracil phosphoribosyltransferase
5-FU: 5-Fluorouracil
DO: Dissolved oxygen
