# Supplementary material for: A facile and robust T7-promoter-based high-expression of heterologous proteins in Bacillus subtilis
Source: Bioresour Bioprocess. 2022 May 18;9(1):56. doi: 10.1186/s40643-022-00540-4 (PMC10991129; doi:10.1186/s40643-022-00540-4)
Supplement: Supplementary file 1 — Additional file1: Figure S1. The standard curve of GFP protein concentration and its fluorescence intensity. Figure S2. SDS-PAGE analysis of IMP of B. subtilis SCK22/pHT7-IMP in a 5-L fermenter over time. Lanes: M, protein markers; T, the cell lysate; S, the supernatant of the cell lysate. Figure S3. SDS-PAGE analysis of expression of 4-α-glucanotransferase (4GT) from Thermococcus litoralis in E.coli BL21(DE3). Lanes: M, protein markers; T, the cell lysate; S, the supernatant of the cell lysate. Table S1. Primers for gene knockout. [file 40643_2022_540_MOESM1_ESM.docx]

Additional file 1 Information

A facile and robust T7-promoter-based high-expression of heterologous proteins in *Bacillus subtilis*

Jing Ye^1 &^, Yunjie Li^2 &^, Yuqing Bai^2^, Ting Zhang^2^, Wei Jiang^2^, Ting Shi^2 *^, Zijian Wu^1 *^,

Yi-Heng P. Job Zhang^2 *^

^1^ Tianjin Key Laboratory of Food Science and Biotechnology, College of Biotechnology and Food Science, Tianjin University of Commerce, Tianjin, China

^2^ Tianjin Institute of Industrial Biotechnology, Chinese Academy of Sciences, 32 West 7th Avenue, Tianjin Airport Economic Area, Tianjin, 300308, China

& Equal contributors.

* Corresponding authors:

Y.-H. P. Zhang (ORCID ID: 0000-0002-4010-2250), email: zhang_xw@tib.cas.cn;

Zijian Wu (ORCID ID: 0000-0001-7093-3023), email: wzjian@tjcu.edu.cn;

Ting Shi (ORCID ID: 0000-0003-1936-2944), email: shi_ting@tib.cas.cn.


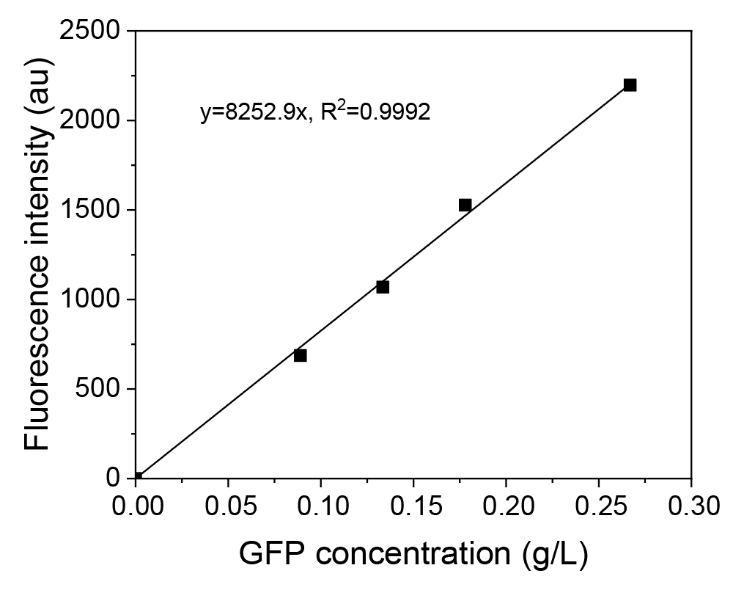


**Figure S1.** The standard curve of GFP protein concentration and its fluorescence intensity.


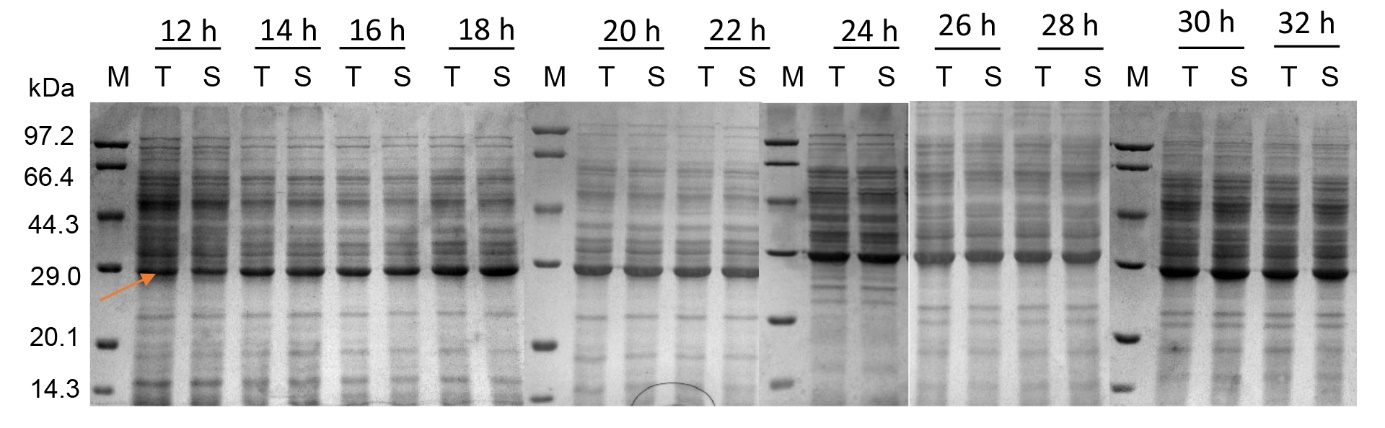


**Figure S2.** SDS-PAGE analysis of IMP of *B. subtilis* SCK22/pHT7-IMP in a 5-L fermenter over time. Lanes: M, protein markers; T, the cell lysate; S, the supernatant of the cell lysate.


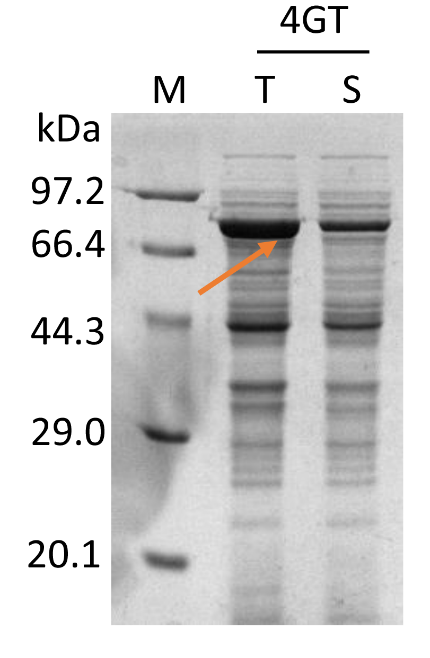


**Figure S3**. SDS-PAGE analysis of expression of 4-alpha-glucanotransferase (4GT) from *Thermococcus litoralis* in *E.coli* BL21(DE3). Lanes: M, protein markers; T, the cell lysate; S, the supernatant of the cell lysate.

**Table S1 Primers for gene knockout.**

|  | **Primers** | **Oligo Sequences (5’→3’)** |
| --- | --- | --- |
| **Primers used in construction of pSS-uppF-cm-uppR** | | |
| pSS plasmid backbone | P21 | ccatatccaccatgaagtaagcgcaagcttggcactggccgtcgttttac |
|  | P22 | cttaaacgttcatcaatggaagagaggggtaccgagctcgaattcgtaat |
| *upp*F (contain DR region) | P23 | gtaaaacgacggccagtgccaagcttgcgcttacttcatggtggatatgg |
|  | P24 | tctagagtcgacgtcgacctgcagtgtcccatcaacaattacacacttctattgattctacaaaaaaagacattgagtttcaagaacatcgtcaaaaaac |
| chloramphenicol resistance gene (cm) | P25 | gttttttgacgatgttcttgaaacttgtcccatcaacaattacacacttctattgattctacaaaaaaagacattgctgcaggtcgacgtcgactctaga |
|  | P26 | ctttacgtctccacagaatgttcatgtcccatcaacaattacacacttctattgattctacaaaaaaagacattgttataaaagccagtcattaggccta |
| *upp*R (contain DR region) | P27 | taggcctaatgactggcttttataacaatgtctttttttgtagaatcaatagaagtgtgtaattgttgatgggacatgaacattctgtggagacgtaaag |
|  | P28 | attacgaattcgagctcggtacccctctcttccattgatgaacgtttaag |
| **Primers used in construction of pDG1730-P43-T7 RNAP** | | |
| pDG1730 plasmid backbone (contain DR region) | P29 | ctacaaaaaaagacattgagtttcaagaacatgatccccctatgcaagggtttattg |
|  | P30 | agagtcggacttcgcgttcgcgtaatcgacatggatgagcgatgatgata |
| *upp* | P31 | caataaacccttgcatagggggatcatgttcttgaaactcaatgtctttttttgtag |
|  | P32 | gttaatcgtgttcattttgaattcctccttttggtaccgctatcactttatattttacataatcgcgcgctttttttcacgcccatttctaaaaatgtaaaataaatgtaagaattttcagccacgatcagaccagtttttaatttgtgtgtttccatgtgtccagtttggaatggatcctctagagtcgacctgcagg |
| P_43_-T7RNAP (contain DR region) | P33 | cctgcaggtcgactctagaggatccattccaaactggacacatggaaacacacaaattaaaaactggtctgatcgtggctgaaaattcttacatttattttacatttttagaaatgggcgtgaaaaaaagcgcgcgattatgtaaaatataaagtgatagcggtaccaaaaggaggaattcaaaatgaacacgattaac |
|  | P34 | tatcatcatcgctcatccatgtcgattacgcgaacgcgaagtccgactct |
| **Primers used in construction of pSS-spoIIACF-upp-cm-spoIIACR** | | |
| pSS plasmid backbone | P35 | gcttcagggtttcagccgtatggtgtaacataaatatatattttaaaaatatc |
|  | P36 | cgatcaatatgtggccatgcatgaggaattcgtaatcatggtcatagctg |
| *spoIIAC*F (contain DR region) | P37 | gatatttttaaaatatatatttatgttacaccatacggctgaaaccctgaagc |
|  | P38 | tccttttttaaaagtcaatattactaacaaatctccttaattacaaagcg |
| *upp*-*cm* | P39 | cgctttgtaattaaggagatttgttagtaatattgacttttaaaaaagga |
|  | P40 | ctgcagactagccatccgtaacaaatctccttaattacaaagcgctttgctttttgataagtgctttgttaagcgaatcgttgttgagctcggtacccggggatcctct |
| *spoIIAC*R (contain DR region) | P41 | agaggatccccgggtaccgagctcaacaacgattcgcttaacaaagcacttatcaaaaagcaaagcgctttgtaattaaggagatttgttacggatggctagtctgcag |
|  | P42 | cagctatgaccatgattacgaattcctcatgcatggccacatattgatcg |
| **Primers used in construction of pSS-srfACF-upp-cm-srfACR** | | |
| pSS plasmid backbone | P43 | cgttgatttcagttccatatggctgatggtaacataaatatatattttaaaaatatcc |
|  | P44 | ggcacatgttcctgctgtcacaaacgaattcgtaatcatggtcatagctg |
| *srfAC*F (contain DR region) | P45 | ggatatttttaaaatatatatttatgttaccatcagccatatggaactgaaatcaacg |
|  | P46 | cttttttaaaagtcaatattactatgtttcgctcccgccttctgtaattccc |
| *upp*-*cm* | P47 | gggaattacagaaggcgggagcgaaacatagtaatattgacttttaaaaaag |
|  | P48 | cttcatttatgaaaccgttacggtttgtgtatgtttcgctcccgccttctgtaattcccttgcgttttattttaaattctcctcaagcatggagctcggtacccggggatcctcta |
| *srfAC*R (contain DR region) | P49 | tagaggatccccgggtaccgagctccatgcttgaggagaatttaaaataaaacgcaagggaattacagaaggcgggagcgaaacatacacaaaccgtaacggtttcataaatgaag |
|  | P50 | cagctatgaccatgattacgaattcgtttgtgacagcaggaacatgtgcc |

Note: The red part represents the DR region sequence.
